# Sequence analysis of the potato aphid *Macrosiphum euphorbiae* transcriptome identified two new viruses

**DOI:** 10.1371/journal.pone.0193239

**Published:** 2018-03-29

**Authors:** Marcella A. Teixeira, Noa Sela, Hagop S. Atamian, Ergude Bao, Ritu Chaudhary, Jacob MacWilliams, Jiangman He, Sophie Mantelin, Thomas Girke, Isgouhi Kaloshian

**Affiliations:** 1 Department of Nematology, University of California, Riverside, California, United States of America; 2 Department of Plant Pathology and Weed Research, Volcani Center, Bet Dagan, Israel; 3 Graduate Program in Computer Science and Engineering, University of California, Riverside, California, United States of America; 4 Department of Botany and Plant Sciences, University of California, Riverside, California, United States of America; 5 Institute for Integrative Genome Biology, University of California, Riverside, California, United States of America; 6 Center for Infectious Disease Vector Research, University of California, Riverside, California, United States of America; Louisiana State University, UNITED STATES

## Abstract

The potato aphid, *Macrosiphum euphorbiae*, is an important agricultural pest that causes economic losses to potato and tomato production. To establish the transcriptome for this aphid, RNA-Seq libraries constructed from aphids maintained on tomato plants were used in Illumina sequencing generating 52.6 million 75–105 bp paired-end reads. The reads were assembled using Velvet/Oases software with SEED preprocessing resulting in 22,137 contigs with an N50 value of 2,003bp. After removal of contigs from tomato host origin, 20,254 contigs were annotated using BLASTx searches against the non-redundant protein database from the National Center for Biotechnology Information (NCBI) as well as IntereProScan. This identified matches for 74% of the potato aphid contigs. The highest ranking hits for over 12,700 contigs were against the related pea aphid, *Acyrthosiphon pisum*. Gene Ontology (GO) was used to classify the identified *M*. *euphorbiae* contigs into biological process, cellular component and molecular function. Among the contigs, sequences of microbial origin were identified. Sixty five contigs were from the aphid bacterial obligate endosymbiont *Buchnera aphidicola* origin and two contigs had amino acid similarities to viruses. The latter two were named Macrosiphum euphorbiae virus 2 (MeV-2) and Macrosiphum euphorbiae virus 3 (MeV-3). The highest sequence identity to MeV-2 had the Dysaphis plantaginea densovirus, while to MeV-3 is the Hubei sobemo-like virus 49. Characterization of MeV-2 and MeV-3 indicated that both are transmitted vertically from adult aphids to nymphs. MeV-2 peptides were detected in the aphid saliva and only MeV-2 and not MeV-3 nucleic acids were detected inside tomato leaves exposed to virus-infected aphids. However, MeV-2 nucleic acids did not persist in tomato leaf tissues, after clearing the plants from aphids, indicating that MeV-2 is likely an aphid virus.

## Introduction

Aphids (Hemiptera: Aphididae) are among the most destructive agricultural insect pests worldwide [1]. They have a short generation time often resulting in vast population expansion during a growing season. Aphids damage their host plants directly through their feeding activity and indirectly by transmitting viruses or supporting the growth of saprophytic fungi such as sooty mold. Aphids are phloem feeders and remove phloem sap, which might otherwise be used for plant growth and reproduction. Moreover, during the feeding process, they inject saliva that can be phytotoxic and contains effectors which modulate plant defenses and may thus predispose the plant to other diseases [2], [3]. Indirect damage caused by plant viruses transmitted by aphids far exceed the aphid’s direct impact on crops [4, 5].

Aphids have complex life cycles, comprising of both sexual and asexual (parthenogenetic) modes of reproduction and wing dimorphism [6, 7]. In addition, they show high diversity in terms of host range and host plant specialization. Moreover, they possess a diverse symbiont community including the mutualistic obligate bacterial endosymbiont *Buchnera aphidicola* that is essential for aphid reproduction and survival [8]. Aphids may also harbor secondary or facultative symbionts, a subset of which are believed to contribute to aphid host range [9]. In addition, aphids establish complex relationships with their plant hosts. Recent studies have shown that aphids produce effectors that modulate host defense responses [2, 10, 11]. The unusual biology of aphids makes them ideal models for the study of several biological processes that are not readily studied in other genetic model systems. Some of these aphid-associated characteristics are expected to be the result of unique sets of genes found in this genus.

The potato aphid *Macrosiphum euphorbiae* belongs to the subfamily Aphidinae [12]. *M*. *euphorbiae* infests many plant species including those from the Solanaceae such as potato and tomato and transmits a number of plant viruses [13]. In tomato, resistance to this aphid is mediated by the *Mi-1* gene that encodes a nucleotide-binding leucine-rich repeat protein [14, 15]. Both *Mi-1*-virulent and avirulent *M*. *euphorbiae* isolates exist in nature [16, 17]. The transcriptome of one *Mi-1* avirulent *M*. *euphorbiae* isolate was generated and used for the identification of the salivary proteome [18, 19]. Using these transcriptome and secretome, several aphid effectors modulating plant immune responses were identified [11, 19, 20]. In addition to these effectors, these resources identified the chaperonin GroEL from the *B*. *aphidicola* endosymbiont as the first aphid-associated molecular pattern to trigger plant immunity [18]. Moreover, analysis of this transcriptome identified a novel virus, the Macrosiphum euphorbiae virus 1 (MeV-1), belonging to the family Flaviviridae with single-stranded RNA genomes [21].

In recent years, genomes of a few aphids have been sequenced and the transcriptomes of additional aphid species have been published [2, 22–30]. In this study, we describe the potato aphid transcriptome. This transcriptome was generated from 128 giga bases of high-quality *M*. *euphorbiae* sequence information using Illumina technology and was *de novo* assembled using the SEED-VELVET/Oasis approach [31, 32]. Based on Gene Ontology (GO) analysis the contigs were assigned to diverse molecular function and biological process categories suggesting a comprehensive representation of the *M*. *euphorbiae* transcriptome. Interestingly, we identified two contigs with homologies to two new viruses and named them MeV-2 and MeV-3. We discovered that peptides of one of these viruses, MeV-2, were detected in the proteome of the *M*. *euphorbiae* saliva and that MeV-2 is secreted into the aphid host plant.

## Materials and methods

### Plants and aphid colonies

Tomato cultivar (cv.) UC82B (*mi-1*/*mi-1*) and near isogenic cv. Motelle (*Mi-1/Mi-1*) and cv. Moneymaker (*mi-1*/*mi-1*) were grown in UC mix II (agops.ucr.edu/pdfs/soil_mix_recipes.pdf) in a growth room at 24^°^C with 16h light/8h dark. A colony of potato aphid (*M*. *euphorbiae*) isolate WU11, acquired from France, was reared parthenogenetically on the susceptible tomato cv. UC82B. Aphids were maintained inside insect cages in a pesticide-free greenhouse at 22–26°C.

Samples of other *M*. *euphorbiae* populations stored in RNAlater (Ambion) were obtained from Canada and the Netherlands.

### Aphid material for library construction

Age-synchronized, one-day-old, adult aphids were generated as described previously [33]. About 200 one-day-old adult aphids were exposed to resistant Motelle for 12 h and 24 h or to susceptible Moneymaker tomato plants for 24 h. An additional 200 one-day old adult aphids were subjected to starvation for 24 h in a Petri dish. Mixed stage aphids were also collected, from the colony reared on the susceptible tomato cv. UC82B.

### RNA extraction, library construction and sequencing

A total of 5 libraries were prepared. For the biotic stress conditions, three libraries were prepared from 200 age-synchronized one day-old adult aphids either exposed to cv Motelle or Moneymaker tomato. For the abiotic stress, a single library was prepared from 200 age-synchronized, one day-old adult aphids. In addition, a single library was prepared from the mixed aphid developmental stages maintained on susceptible tomato. For Illumina library preparation, RNA was extracted using the RNeasy Midi kit according to the manufacturer’s recommendation (Qiagen). Twenty μg of RNA was treated with DNase I enzyme (New England BioLabs) followed by phenol-chloroform extraction and isopropanol precipitation. The RNA quality and integrity were evaluated using an Agilent 2100 BioAnalyzer (Agilent Technologies).

RNA-Seq libraries were prepared for high-throughput sequencing on the Illumina Cluster Station and Genome analyzer as described by [34]. In brief, mRNA was isolated from 4 μg of the DNase-treated total RNA using Sera-mag Magnetic oligo(dT) beads and fragmented with divalent cations under elevated temperatures. The cleaved mRNA fragments were copied into first- and second-strand cDNA using random primers. The overhangs were converted into blunt ends using T4 DNA polymerase and Klenow DNA polymerase, followed by the addition of an “A” base to the 3’ end of the blunt phosphorylated cDNA fragments. Adapters were ligated to the ends of the cDNA fragments, purified on a gel and 300 bp templates selected for downstream enrichment by PCR using primers complementary to the adapter sequences. The size, purity and concentration of the prepared library were evaluated by running 1 μl on a 2% agarose gel. To assess the diversity of the library, 1 μl of the library was cloned into the Zero Blunt TOPO vector following the manufacturer’s recommendation (Invitrogen) and 10 clones were sequenced. TBLASTx searches identified distinct sequences for each clone within each library suggesting that the libraries were not biased.

The RNA-Seq library from mixed aphid developmental stages was run on two flowcell lanes, while the remaining four libraries were combined together and run on a single flowcell lane.

Paired-end 75 or 105 nucleotide-long sequencing was performed with the Illumina Cluster Station and Genome Analyzer II at the Institute for Integrative Genome Biology, University of California, Riverside.

### *de novo* assembly of reads and annotation

Data from Illumina Genome Analyzer II sequencing runs were processed using the Illumina pipeline version 1.4 to generate sequencing reads, base-call quality scores, and remove low quality reads. The sequence data generated were deposited in National Center for Biotechnology Information’s (NCBI) Sequence Read Archive SRA) (SRP029202), accession number SRX339176. The reads were assembled by first applying SEED (1.2.1) preprocessing to cluster near identical RNA reads followed by Velvet/Oases (1.0.15/0.1.18) to assemble the resulting center reads in each cluster and the initial reads, respectively, as described in Bao *et al*. [31]. The assembled transcriptome was deposited in NCBI (accession number GAOM00000000). The resulting contigs were annotated by BLASTx searches against NCBI’s non-redundant (nr) database (E-value cut off 1e-3) and InterProScan searches [35] against several protein sequence, domain and motif databases. For *Buchnera* sequence annotation, BLASTx searches against the UniProt database were used.

### Virus detection in aphids and tomato leaves

Nucleic acids were isolated from tomato leaflets or aphids using Trizol (Invitrogen) and used for cDNA synthesis using Superscript III reverse transcriptase (Invitrogen) and oligo(dT) primers according to the manufacturer’s recommendations. For single aphid nucleic acid extraction, acrylamide (Fisher) was added as a carrier before precipitation. PCR was performed in 25 μl reactions using the following primers: MeV2-F 3’CCGGATGACAAATCCCACGA5’ and MeV2-R 3’AATAGGCGCAGAGATGGACG5’; MeV3-F: TTTTGACTTGACCTATGGTTCCCTT and MeV3-R: AGCCAATTTAGTACCATCACTACGT. PCR conditions were 94C for 5 min, followed by 35 cycles of 94C for 30 sec, 60C (MeV2) or 53C (MeV3) for 30 sec, 72C for 30 sec and a final cycle at 72C for 3 min. The aphid ribosomal protein L27 (*RpL27*) [20] and the tomato ubiquitin (*SlUbi3*) [36] were used as control. Products were separated by electrophoresis in 1.2% agarose gels and visualized by ethidium bromide staining.

## Results

### Transcriptome sequencing and assembly

To maximize the genome coverage of the *M*. *euphorbiae* expressed genes in our experimental material, we prepared RNA-Seq libraries representing transcripts from mixed aphid developmental stages as well as aphids exposed to various biotic or abiotic stresses. Five libraries were constructed and run in three flowcell lanes generating a total of 52.6 million paired-end reads.

We applied SEED (1.2.1) [31] to cluster the RNA reads, and then used Velvet/Oases (1.0.15/0.1.18) [32] to assemble the resulting center reads in each cluster and the initial reads, respectively. We used the VelvetOptimiser (2.1.7) tool to find the best k-mer length for Velvet/Oases between 19 and 71. The Velvet/Oases with SEED pre-processing generated 22,137 contigs with an N50 value of 2,003bp. The N50 value is the contig length where 50% of the entire assembly is contained in contigs of at least this value.

### Annotation and gene ontology assignments

**Fig 1:**
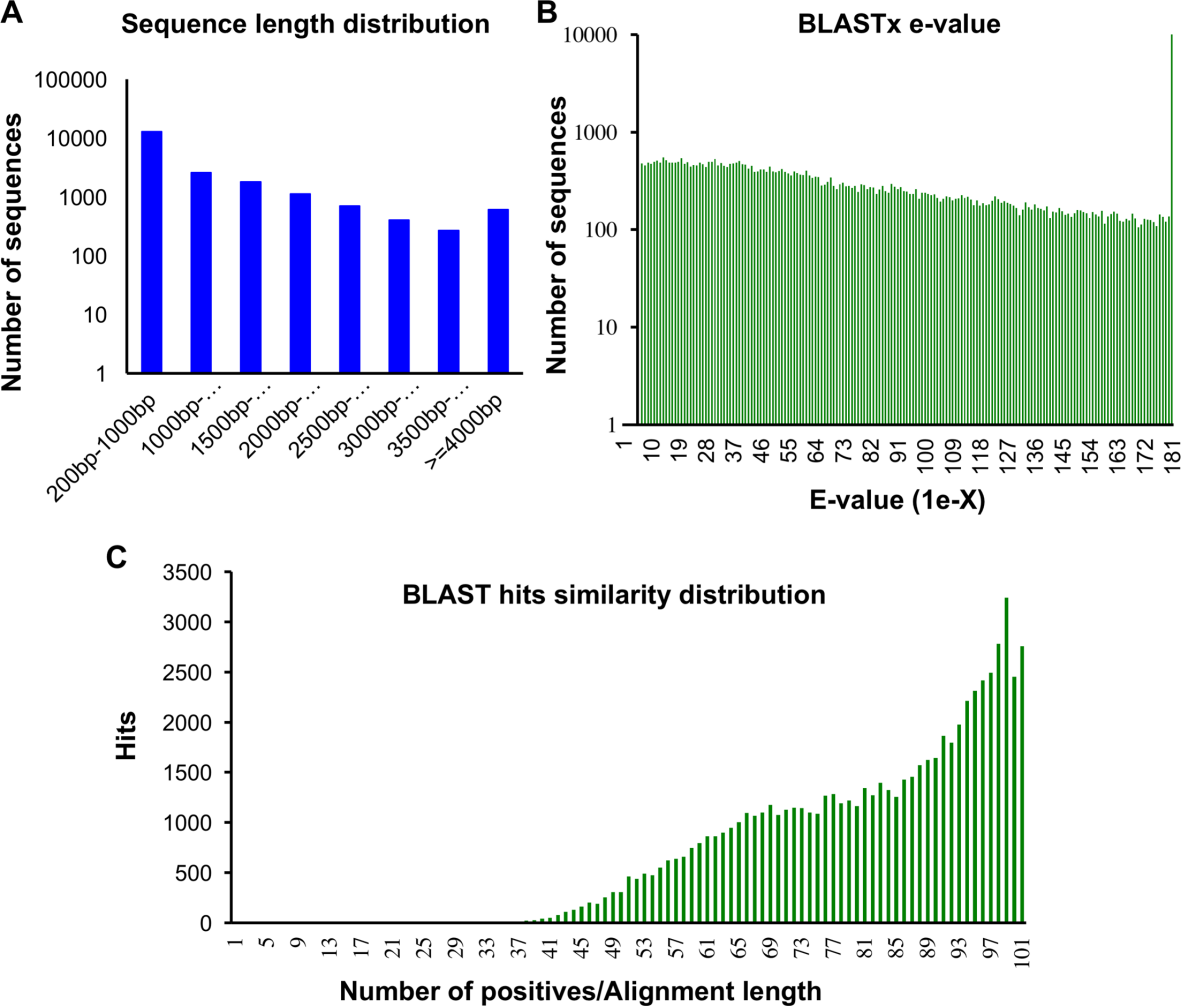
Characterization of the *de novo* assembly of the *Macrosiphum euphorbiae* transcriptome. Distributions of (A) Sequence length (B) BLASTx E-values and (C) Sequence BLASTx hits similarity.

The contigs were annotated by BLASTx searches against the NCBI’s NR protein database and InterProScan searches on different protein databases (Fig 1; S1 Table) [35]. About 8.2% (1,818/22,137) of the assembled sequences identified as from tomato origin were considered contaminants and consequently excluded from downstream analyses as well as the potato aphid transcriptome we submitted to NCBI. Moreover, BLASTx analysis against the UniProt database revealed that 65 contigs originated from the aphid endosymbiont *Buchnera* (S2 Table). These sequences were also excluded from the potato aphid transcriptome.

**Fig 2:**
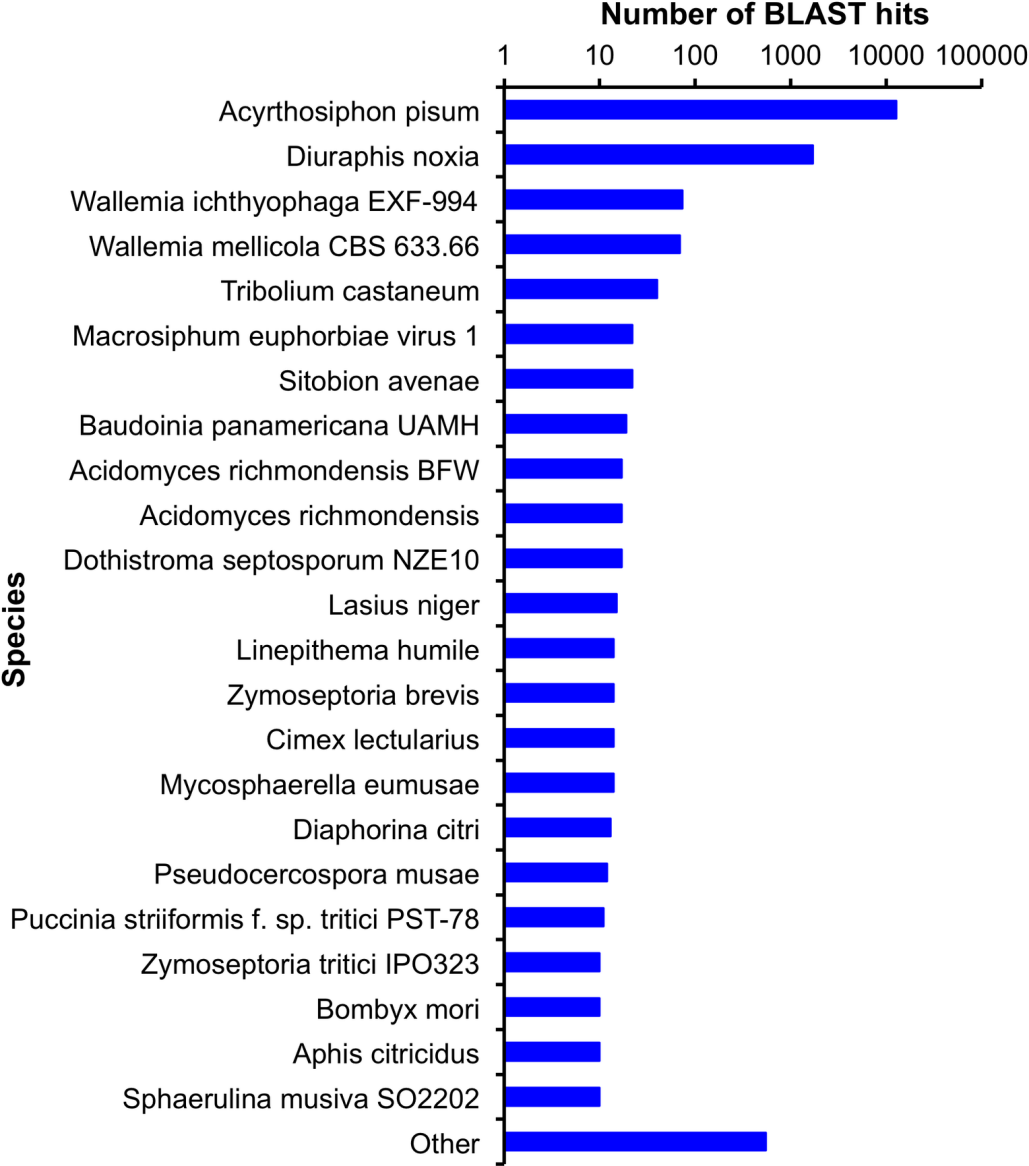
The *M*. *euphorbiae* transcriptome top hits species distribution. Data obtained using BLASTx analysis in NCBI’s non-redundant protein database.

Of the remaining assembled contigs, over 82% were greater than 300 bp in length (Fig 1A). About 74% (15,139/20,254) showed sequence similarity to proteins in NCBI’s nr protein database (E < 1e^-3^) (Fig 1B and 1C). The sequences were annotated based on their matches to the nr database by Blast2go software [37]. In addition, the contigs were translated into protein and scanned with InterProScan against several protein databases (S2 Table). The top blast-hits for the known contigs showed 84.4% (12,781/15,139) matches with *A*. *pisum* sequences and 11.2% (1,704/15,139) matches with the Russian wheat aphid, *Diuraphis noxia* (Fig 2).

**Fig 3:**
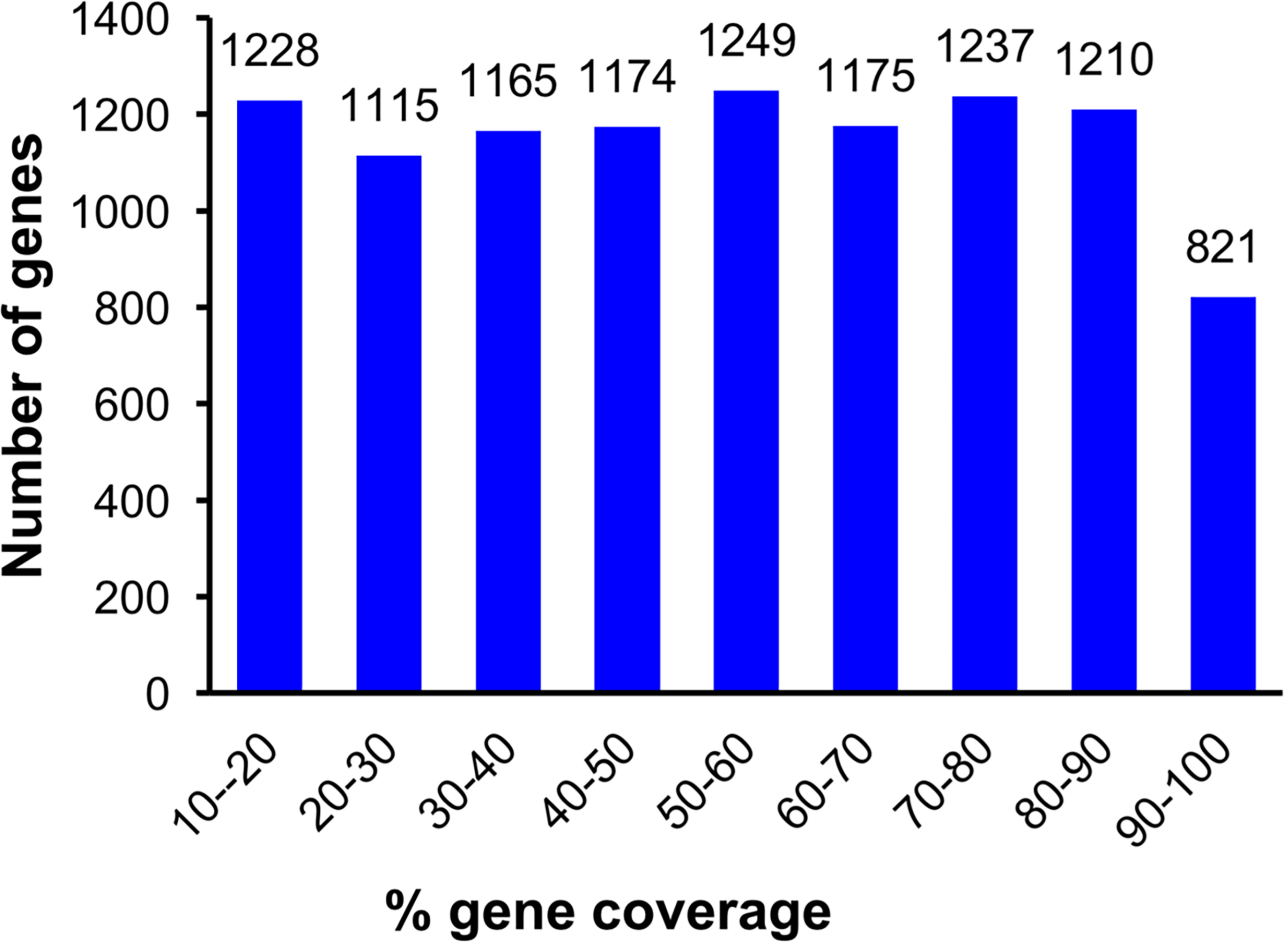
*Acyrthosiphon pisum* gene coverage by *M*. *euphorbiae* contigs. Histogram showing the coverage of *A*. *pisum* predicted genes by the *de novo* assembled *M*. *euphorbiae* transcriptome.

Finally the 12,781 contigs were aligned to the predicted *A*. *pisum* transcriptome (ACYPI mRNA v2.1) in AphidBase 2.1. More than 6,800 of the *A*. *pisum* transcripts had over 40% coverage by their corresponding *M*. *euphorbiae* contigs (Fig 3).

**Fig 4:**
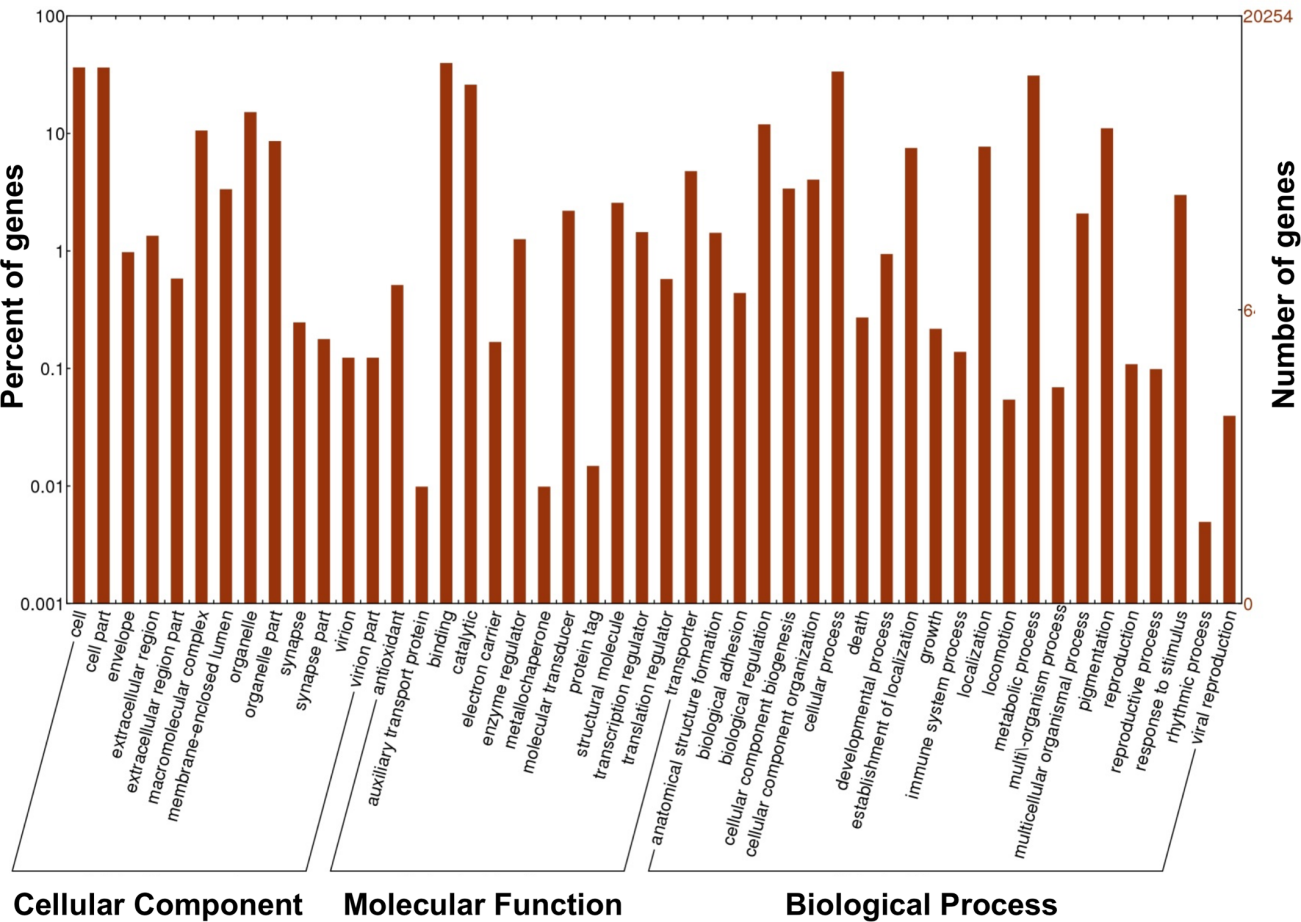
Histogram of *the M*. *euphorbiae* transcriptome gene ontology (GO) classification. GO level 2 descriptions of the indicated three main categories. The visualization of GO distribution was done with WEGO tool (http://wego.genomics.org.cn/).

To classify the functions of *M*. *euphorbiae* contigs, we used the Blast2GO software to map the contigs GO terms. The contigs were categorized into 43 functional groups within the three main ontologies, defined as cellular components, molecular function, and biological process (Fig 4). Within the cellular component ontology category, a high proportion of genes was associated with the terms cell (7,388 genes, 36.5%) and cellular parts (7,367 genes, 36.4%). For the molecular function ontology, a high percentage of genes was assigned to binding (8,049 genes, 39.7%) and catalytic activity (5,269 genes, 26%) categories, while the most abundant biological process terms were cellular processes (6,812 genes, 31.1%) and metabolic processes (6,297 genes, 31.1%) (Fig 4).

**Fig 5:**
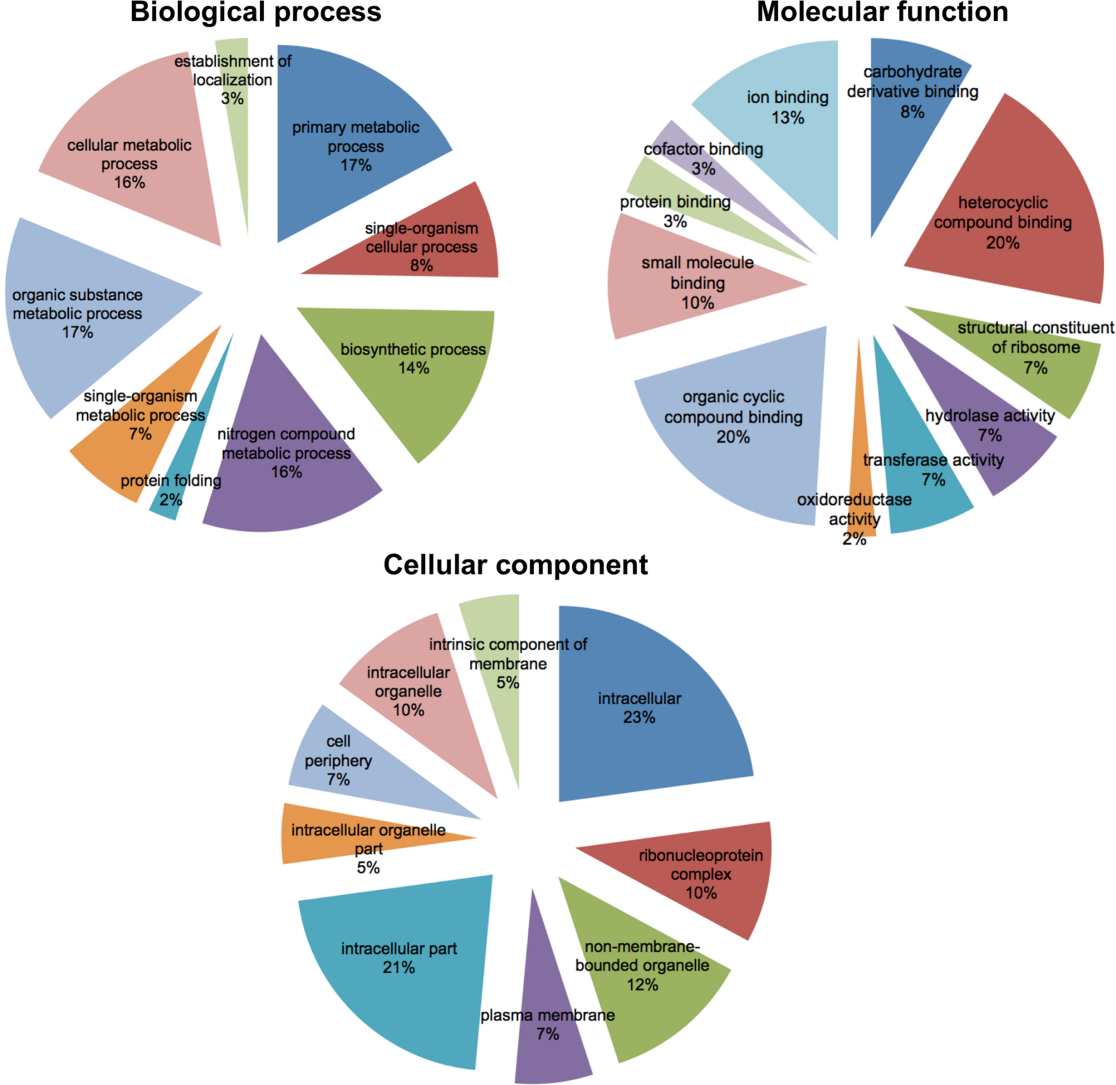
Gene ontology (GO) classification of the endosymbiont *Buchnera aphidicola*. GO level 3 description of *Buchnera* transcripts identified among the *M*. *euphorbiae* transcriptome.

GO term enrichment analysis of the *Buchnera* sequences revealed within the biological process ontology, the highest representations for primary metabolic processes (11 genes, 17%) and organic substance metabolic processes (11 genes, 17%) (Fig 5). Within the molecular function ontology, the most abundant GO terms were heterocyclic compounds (13, 20%) and organic cyclic compounds binding (13, 20%); and for the cellular component ontology it was intracellular (15 genes, 23%) and intracellular part (14 genes, 21%) (Fig 5).

### Contigs of viral origins

**Fig 6:**
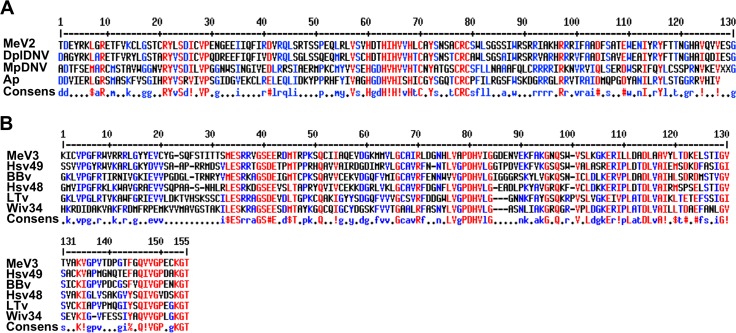
Line up of MeV-2 and MeV-3 sequences with their respective homologous sequences. (A) Amino acid translation of MeV-2 transcript (GAMO01012456.1; Me_WB16380), with its related virus proteins including: Dysaphis plantaginea DNV (DplDNV; ACI01073.1); Myzus persicae DNV (MpDNV; NP_874375.1); and *Acyrthosiphum pisum* uncharacterized protein (Ap; XP-016664361.1). (B) Amino acid translation of MeV-3 (GAOM01011582.1; Me_WB14511) with its related virus proteins including: Hubei sobemo-like virus 49 (Hsv49; APG75768.1), Braid Burn Virus (BBv; AMO03213.1), Hubei sobemo-like virus 48 (Hsv48; APG75765.1), La Tardoire virus (LTv; AMO03214.1), and Wuhan insect virus 34 (Wiv34; APG75723.1). Amino acids in red indicate high consensus, blue low consensus and black neutral.

In addition to MeV-1 (KT309079), which was previously identified as a novel aphid virus [21], two of the assembled sequences (GAMO01012456.1/Me_WB16380 and GAOM01011582.1/Me_WB14511) showed sequence similarities to viral sequences. The Me_WB16380 contig is 2,668 nucleotides in length. BLASTx searches against NCBI-NR revealed top hits to Dysaphis plantaginea densovirus (DplDNV) (ACG50804.1) (36% coverage; E = 2e-71) and to a predicted protein from *A*. *pisum* (LOC100575585; XP_016656124.1) (12% coverage; E = 9e-10) as well as to a putative nonstructural protein NS-1 of Myzus persicae densovirus (MpDNV) (NP_874375.1) (14% coverage; E = 4e-08). The translated proteins of these sequences indicated presence of nonstructural viral protein sequences (Fig 6A). Therefore, it is likely that Me_WB16380 is of viral origin. Therefore, we named this virus Macrosiphum euphorbiae virus 2 (MeV-2).

The second contig, Me_WB14511, is 478 nucleotides in length and BLASTx searches against NCBI-NR revealed high similarities to the following viruses: Hubei sobemo-like virus 49 (APG75768.1) (99% coverage; E = 4e-45), Braid Burn virus (AMO03212.1), (97% coverage; E = 2e-39), Hubei sobemo-like virus 48 (APG75765.1) (97% coverage; E = 3e-34); La Tardoire virus (AMO03214.1), 97% coverage; E = 9e-32); and to Wuhan insect virus 34 (APG75723.1) (82% coverage; E = 4e-26) (Fig 6B). Based on these high similarities to viral sequences, it is likely that contig Me_WB14511 is also of viral origin and we named this virus Macrosiphum euphorbiae virus 3 (MeV-3).

### Detection of MeV-2 and MeV-3 in *M*. *euphorbiae*

**Fig 7:**
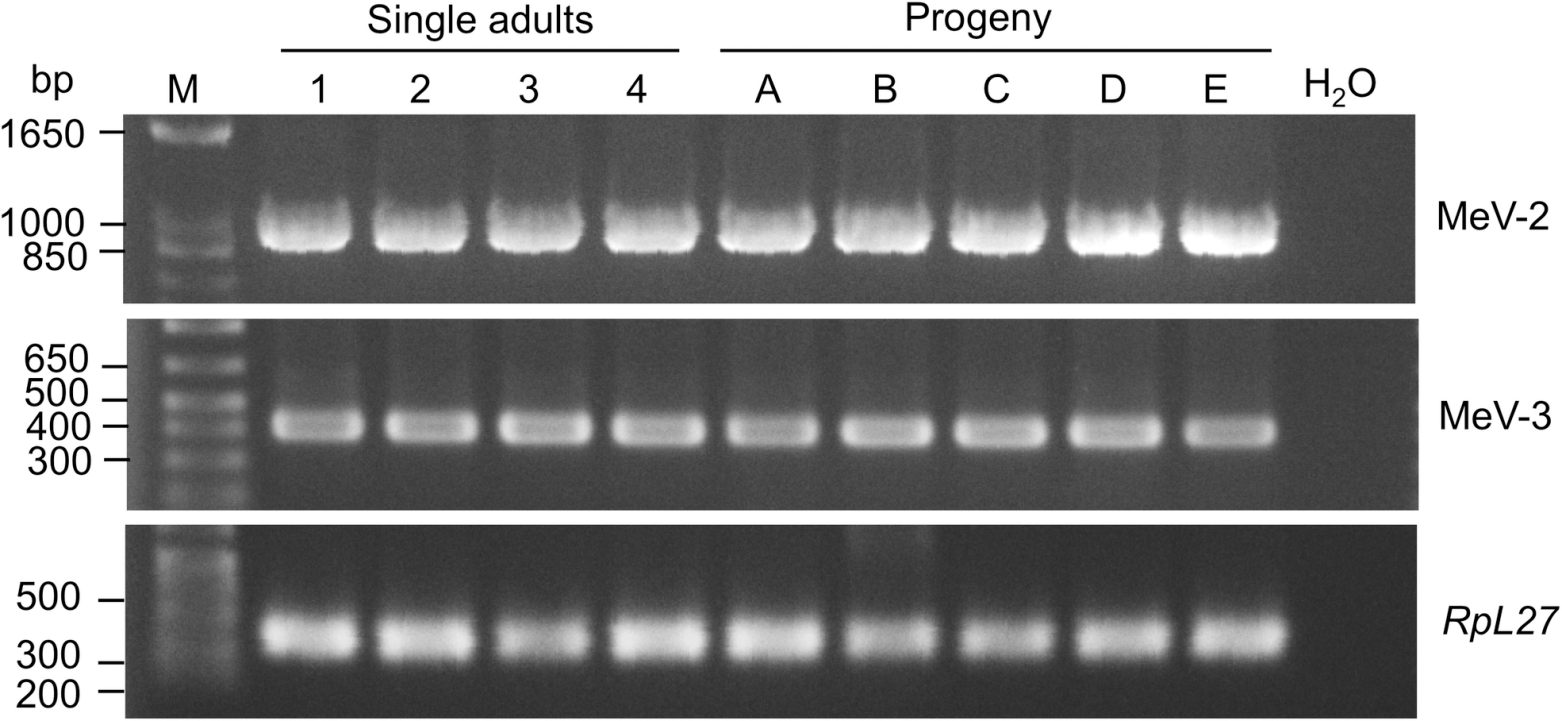
MeV-2 and MeV3 are present in *M*. *euphorbiae* WU11 population and is vertically transmitted to progeny. Aphid nucleic acids were used in RT-PCR for MeV-2 and MeV-3 detection. For evaluation of vertical transmission, first instar nymphs were collected while being laid from adult aphids, before touching tomato leaflets, using a brush and transferred to a naïve tomato plant. One week later, when nymphs had molted into adults, single aphids were processed for the presence of MeV-2 and MeV-3. Aphid ribosomal gene *RpL27* was used as positive control. M = molecular weight marker.

Based on sequence similarity, MeV-2 belongs to the genus Densovirus with single-stranded (ss) DNA genomes and is likely an aphid virus [38]. To determine the extent of MeV-2 presence in our *M*. *euphorbiae* population, we investigated the presence of this virus in 12 randomly selected individual adult aphids. MeV-2 was detected in all these aphids suggesting that the virus is vertically transmitted from adult aphids to nymphs (Fig 7). To confirm the transovarial transmission of the virus, adult aphids, collected as first instar nymphs from the posterior ends of the mothers while being delivered, and grown on naive tomato plants, were also tested for the presence of the virus. Of the ten aphids tested, all were positive for MeV-2 (Fig 7).

In contrast to MeV-2, MeV-3 belongs to the Luteo-sobemo group of viruses with positive-sense RNA genomes [39]. Recently viruses from the Luteo-sobemo group have been detected from mix insect species from China suggesting MeV-3 is also an arthropod virus [39]. Therefore, we investigated the distribution of MeV-3 in our *M*. *euphorbiae* population. MeV-3 was detected in all four individual adult aphids tested (Fig 7). In addition, the virus was also detected in all aphid progeny collected as first instars from the posterior ends of their mothers suggesting vertical transmission of MeV-3 (Fig 7).

**Fig 8:**
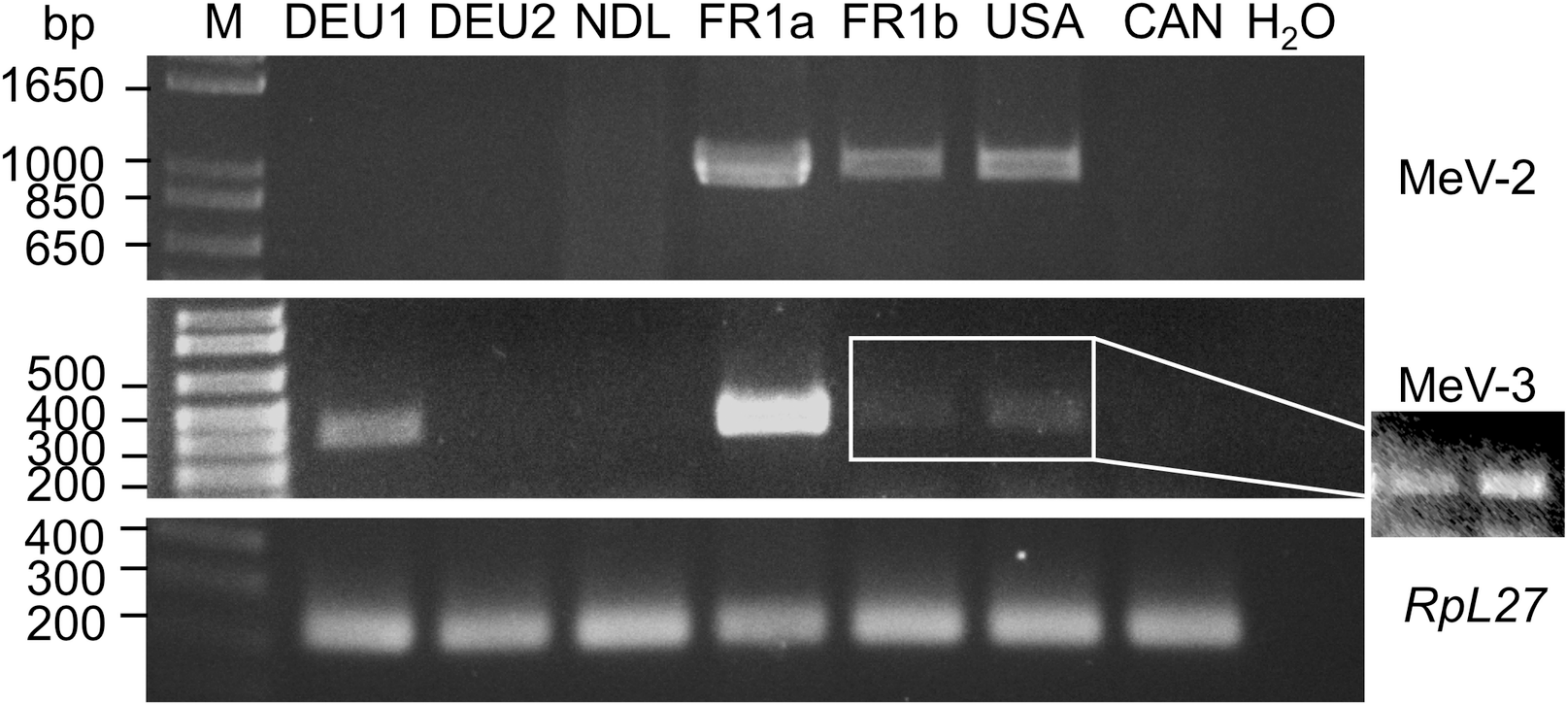
MeV-2 and MeV3 are present in different *M*. *euphorbiae* (*Me*) populations. Nucleic acids from mixed developmental stages of aphids were used in RT-PCR for the detection of MeV-2 and MeV-3. *Macrosiphum euphorbiae* from Germany (DEU), the Netherlands (NDL), France (FR) the United States of America (USA), and Canada (CAN) were used. The population from France is WU11 colony from which the virus was identified. Arabic numerals stand for different aphid populations. FR1a and FR1b colonies are from the same *M*. *euphorbiae* population separated for at least 14 years. Aphid ribosomal gene *RpL27* was used as positive control. M = molecular weight marker. The cropped two lanes of the MeV-3 gel, displays enhanced imaging of the two amplified bands.

The population of *M*. *euphorbiae* WU11 was originally acquired from France. To test the presence of this virus among *M*. *euphorbiae* populations from Europe and north America, *M*. *euphorbiae* isolates were obtained from Germany, the Netherlands, Canada and USA (California). MeV-2 was detected only from the USA population and not from populations from the European countries or Canada (Fig 8). Our aphid that was imported from France has been maintained in the lab for over 14 years. To eliminate the possibility that the virus was introduced to this aphid population (FR1a) while in the lab, we tested the presence of MeV-2 in a sister colony (FR1b) that was imported from the same source in France but kept in a different location in the USA. MeV-2 was also detected in this later aphid population as well (Fig 8).

Using the same *M*. *euphorbiae* populations, the distribution of MeV-3 was also evaluated. MeV-3 was detected from the USA population as well as in the sister colony (FR1b) originating from France albeit at very low titers. In contrast to MeV-2, in addition to the USA population, MeV-3 was also detected from a *M*. *euphorbiae* population from Germany (Fig 8). Interestingly, MeV-3 was not detected in a second *M*. *euphorbiae* population from Germany (Fig 8).

### Detection of MeV2 in the plant host

**Fig 9:**
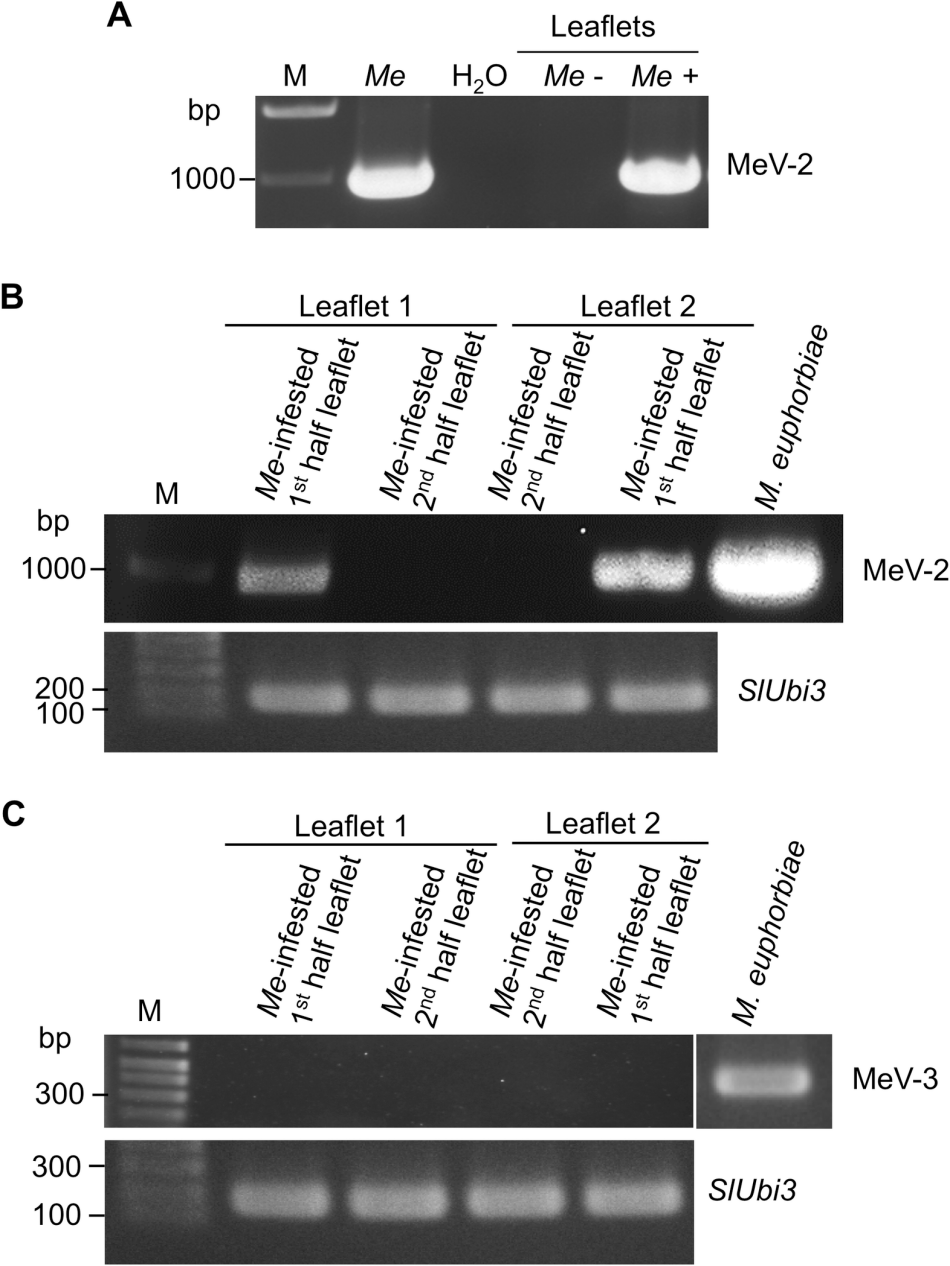
MeV-2 is delivered by *M*. *euphorbiae* (*Me*) into plant tissues during feeding but does not persist in the plant in the absence of the aphid. (A) Nucleic acids isolated from leaves of naïve tomato plants or from plants infested for 2 weeks with MeV-2-infected *M*. *euphorbiae* were used in RT-PCR. (B, C) *M*. *euphorbiae* heavily infested tomato leaves were cleared of the aphids. Leaflets were cut into halves longitudinally through the midrib and the detached half was processed for MeV-2 (B) or MeV-3 (C) detection. The second half of the leaflet was left attached to the plant, free of aphids, for 14 additional days before processing. *SlUbi3* was used as a positive control. M = molecular weight marker.

Inspecting the *M*. *euphorbiae* salivary secretome [18, 19], we identified peptides belonging to MeV-2 but not to MeV-3 in the saliva of this aphid. Detection of peptides derived from MeV-2 proteins in *M*. *euphorbiae* saliva suggested that the virus is delivered into plant tissues during aphid feeding. To test for the presence of MeV-2 in plant tissues, tomato leaflets heavily infested with MeV-2 infected *M*. *euphorbiae* were used. Using RT-PCR, MeV-2 was detected in leaves of aphid-infested plants but not in leaves of control naïve plants not exposed to aphids (Fig 9A). We also tested the dynamic of MeV-2 within a tomato leaflet. Heavily infested tomato plants were cleared from MeV-2-infected aphids, and leaflets were cut through the mid vein collecting half of the leaflet and leaving the second half attached to the plant. Analyzing the first halves of the leaflets for the presence of MeV-2, the virus could be detected by PCR in these infested halve leaflets (Fig 9B). However, two weeks later, no MeV-2 was detected in the second halves of these leaflets (Fig 9B).

MeV-3 could be present at low titer in the aphid saliva and be below the mass spectrometry detection limit. To confirm that MeV-3 is not secreted into the plant, we also tested the presence of MeV-3 in the aphid-infested tomato leaves using RT-PCR. MeV-3 was not detected in the infested tomato leaves confirming the proteomics analysis of the aphid saliva (Fig 9C).

## Discussion

For organisms for which full genome sequences are not available, transcriptome sequencing and *de novo* assembly provides an alternative to build genomics resources as a basis for future studies. High-throughput sequencing technologies, with deep coverage at base level resolution, ease of library preparation and requirement for low quantity of total RNA as starting material, made possible the inclusion of sequencing in studies aimed at finding answers to numerous biological questions. Moreover, transcriptome sequencing addresses the expressed part of the genome, which cannot be unequivocally predicted from the genome sequence alone. Upon genome sequence availability, the transcriptome sequences represent a valuable resource for accurate gene finding, including the identification of splicing patterns. The characterization of a comprehensive set of expressed genes from an organism requires the construction of libraries from different tissues and biological conditions. With this in mind, we used the Illumina technology to sequence libraries derived from mixed developmental stages of *M*. *euphorbiae* and *M*. *euphorbiae* exposed to different biotic or abiotic stresses. These libraries were developed before sequence-tagging technology for multiplexing was commercially available. The libraries were mixed before sequencing as our interest was to build the transcriptome resource for this aphid. Therefore, transcripts associated with specific biotic and abiotic treatments could not be inferred from this study. However, the different biotic and abiotic treatments and aphid developmental stages used are expected to provide a wide diversity of gene expression patterns and consequently a more comprehensive transcriptome set to be derived. The reads were *de novo* assembled into 22,137 contigs (N50 = 2,130 bp) using the SEED/Velvet/Oases approach [31]. Various *de novo* transcriptome assembly algorithms are freely available. Each has advantages and disadvantages and one has to choose among different assemblers the most suitable for the specific application [40]. For this study we chose to apply a method that improves transcriptome assemblies by preprocessing the reads with a clustering approach [31].

Sequences from tomato origin were identified among the contigs. The source of these tomato sequences most likely is the trichomes on tomato leaf surfaces. Since aphids of different developmental stages were collected by carefully brushing the aphids from tomato leaflets, we must have also collected tomato trichomes along with aphids. In addition to tomato sequences, contigs originating from the aphid-associated endosymbiont *Buchnera* were also identified among the aphid transcriptome. The genome of *Buchnera* is AT-rich, with about 73% of AT-rich regions [41]; therefore, some of its sequences must have been captured during the mRNA purification step by oligo(dT) magnetic beads used for the library preparation and were represented among the aphid sequences.

Since *M*. *euphorbiae* is closely related to *A*. *pisum* [12], we used the predicted gene set of the *A*. *pisum* as reference to assess the quality of the contigs assembled in this study. More than 6,800 *A*. *pisum*-predicted transcripts have at least 40% coverage by the *M*. *euphorbiae* transcriptome generated, providing a valuable resource for future gene expression analysis and identifying genes regulated by host-aphid interactions as well as other aphid related processes.

As part of the analysis of the *M*. *euphorbiae* transcriptome, we previously described a new aphid virus, MeV-1, belonging to the positive polarity ssRNA genomes family *Flaviviridae* [21]. Here we describe the discovery of two additional viruses, MeV-2 and MeV-3, in this same aphid transcriptome. The analysis of transcriptome sequences generated by high-throughput sequencing technologies has enabled the discovery of a large number of known and novel viruses from diverse insect species [39, 42–44]. The top blast hits to the newly identified MeV-3 coding sequences all belong to novel viruses recently identified through analysis of high throughput sequences of various insect transcriptomes. The Hubei sobemo-like virus 49 was identified from Odonata [39], Wuhan insect virus 34 was identified from a mixed insect source [39], Braid Burn virus from *Drosophila subsilvestris* [44] and La Tardoire virus from *Scaptodrosophia deflexa* [44] transcriptomes. Most of these viruses have incomplete genomes, their classification is not yet resolved but they seem to be associated with Sobemoviruses which are RNA viruses. The diversity of insects infected with this group of viruses suggests that these novel RNA viruses constitute a group of arthropod infecting viruses.

The MeV-2 contig encodes a nonstructural protein, presenting high homology to previously described aphid Densoviruses such as the rosy apple aphid (*D*. *plantaginea)* DplDNV [45] and green peach aphid (*M*. *persicae*) MpDNV [46]. While infection with either of these viruses negatively affect the aphid hosts, MeV-2 infected *M*. *euphorbiae* do not exhibit any observable pathology. MpDNV infected *M*. *persicae* exhibit abnormal growth and development [46] while the DplDNV infected *D*. *plantaginea* have reduced reproduction rate and wing development in the absence of triggers inducing wing formation, such as crowding or short-day length [45]. Our *M*. *euphorbiae* colony (strain WU11) is infected with at least three viruses with no obvious pathology or unusual phenotypic characteristics. It remains to be seen whether these viruses individually or combined contribute to subtle differences in the aphid biology.

In our *M*. *euphorbiae* colony, MeV-2 and MeV-3 infected aphids seem to be common as the virus was detected from every single aphid tested. In addition, both MeV-2 and MeV-3 are transmitted vertically and likely transovarially since it could be detected in all adult aphids collected as first instar nymphs, while being delivered by their mothers and grown on naïve plants. Vertical transmission seems to be common among aphid Densoviruses as vertical transmission from mother to nymphs have been demonstrated for both DplDNV and MpDNV [45, 46].

Unlike MeV3, both MeV-1 and MeV-2 derived peptides were detected in *M*. *euphorbiae* saliva, indicating that similar to MeV-1, MeV-2 is also delivered through the saliva into plant host tissues [21]. Indeed, MeV-2 nucleotides were detected in tomato leaflets fed on by MeV-2-infected aphids. However, since MeV-2 was not detected in the plant tissues 2 weeks after aphids were cleared from the plants, MeV-2 is not likely to be a plant virus. However, it remains unclear whether MeV-2 can be transmitted horizontally through the plant host to naïve *M*. *euphorbiae* or to other aphid species or to additional piercing-sucking insect species.

Our *M*. *euphorbiae* population (WU11) from which all three viruses (MeV-1, MeV-2 and MeV-3) were identified originated from France and has been reared under greenhouse conditions for about 16 years. Therefore, the presence of MeV-2 and MeV-3 among additional *M*. *euphorbiae* populations was unknown. Evaluating *M*. *euphorbiae* populations, collected from different European and North American geographical locations, showed that these viruses are present only in certain populations from both continents. The presence of these viruses in *M*. *euphorbiae* populations originating from different geographical locations indicates that these virus infections are not likely arisen under laboratory conditions. Moreover, although the *M*. *euphorbiae* populations from Germany and France have been maintained under greenhouse conditions for many years, the population from the USA is relatively new and been in captivity for about a year.

MeV-1, unlike MeV-2 and MeV-3, is present in several *M*. *euphorbiae* populations from different European countries but not from the US or Canada, indicating geographical isolation of MeV-1 infections [21], and the likelihood that infections of *M*. *euphorbiae* by these three viruses occurred independently. Similarly, not all *M*. *euphorbiae* populations tested were infected with both MeV-2 and MeV-3 also indicating independent infections by these two viruses. The prevalence of such cryptic viruses among aphids is not well documents and the study of cryptic insect viruses associated with herbivorous insects is at its infancy [43, 47]. An increasing body of evidence from various organisms [39, 44, 48, 49] combined with our work described here, suggest the potential of additional discoveries of herbivorous insect associated viruses. Considering that the MeV-2 and MeV-3 genome sequences are incomplete and the presence of large number of unknown, with no BLAST hits, and short sequences among the *M*. *euphorbiae* transcriptome, and likely among other herbivorous insect transcriptomes, suggest the likelihood of new virus discoveries. The persistence of the identified viruses in the *M*. *euphorbiae* populations suggests beneficial effects to the insect host. The exciting questions remain as how these viruses contribute to the well-being of their insect host and their role in the insect’s adaptation to plant hosts and to abiotic environmental changes.

## Supporting information

S1 Table Annotation of the *Macrosiphum euphorbiae* transcriptome. **Annotation was performed using** BLASTx analysis against NCBI’s non-redundant protein database and UniProt database.
(XLSX)

S2 Table *Buchnera aphidicola* sequences identified among the *Macrosiphum euphorbiae* transcriptome. Annotation was performed by BLASTx analysis against the UniProt database.
(XLS)
